# Minimum Inhibitory Concentrations of Vancomycin and Daptomycin Against Methicillin-resistant Staphylococcus Aureus Isolated from Various Clinical Specimens: A Study from South India

**DOI:** 10.7759/cureus.6749

**Published:** 2020-01-23

**Authors:** Vinay Kumar Moses, Venkataramana Kandi, Sanjeev Kumar D Rao

**Affiliations:** 1 Microbiology, Government Medical College, Siddipet, IND; 2 Clinical Microbiology, Prathima Institute of Medical Sciences, Karimnagar, IND; 3 Microbiology, MNR Medical College & Hospital, Hyderabad, IND

**Keywords:** staphylococcus aureus, vancomycin, daptomycin, methicillin-resistant staphylococcus aureus, mrsa, s. aureus, minimum inhibitory concentration (mic)

## Abstract

Background

Staphylococci are Gram-positive cocci arranged in clusters. They are colonized in humans and animals. Also, *Staphylococcus aureus* (S. aureus) is frequently associated with various superficial to deep-seated infections in humans. Due to the potential for easy transmission, Staphylococci are associated with both hospital-acquired and community-associated infections. Strains of *S. aureus* resistant to methicillin (MRSA) pose treatment challenges. In such cases, vancomycin is the treatment of choice. Due to the indiscriminate use of vancomycin, recently, we are seeing the emergence of vancomycin-intermediate sensitive *S. aureus* (VISA) and vancomycin-resistant *S. aureus* (VRSA). The present study aims to evaluate the minimum inhibitory concentrations (MICs) of vancomycin and daptomycin among MRSA strains isolated from human clinical specimens

Methods

The study included 115 MRSA isolates collected over 26 months from July 2010 to September 2012. The strains were isolated from pus, urine, wound swabs, catheters, blood, and sputum. The bacteria were acquired from different inpatient and outpatient departments of Prathima Institute of Medical Sciences, Karimnagar, Telangana, India. Kirby-Bauer disk diffusion method using cefoxitin was used to confirm the MRSA isolates. The agar dilution and the Epsilometer method (E-test) were used to test the MICs of MRSA isolates against vancomycin and daptomycin, respectively, by the standard procedures recommended by the clinical laboratory standards institute (CLSI).

Results

Of the 115 *S. aureus* isolates, seven (6.08%) strains were resistant to vancomycin (VRSA) and 53 (46.08%) were found to be VISA using the new CLSI breakpoints. The MIC of the daptomycin was found to be ≤1 µg/ml for all the MRSA isolates.

Conclusion

The study results depicted an increasing trend in the vancomycin MICs among the MRSA isolates. Several tested strains show MICs in the intermediate sensitive range (VISA). The daptomycin was effective against all the MRSA isolates.

## Introduction

*Staphylococcus aureus *(*S*. *aureus*) is a Gram-positive bacterium that is present as normal flora in humans and animals [1]. It is transiently colonized in the nose and on the skin of healthy people [2]. *S*. *aureus* usually causes asymptomatic colonization in healthy people but can result in mild to severe infections in debilitated individuals if the bacteria breach the skin’s defenses [3]. Infections caused by *S*. *aureus *include boils, abscesses, impetigo, cellulitis, folliculitis, carbuncles, scalded skin syndrome, wound infections, and life-threatening illnesses like pneumonia, meningitis, osteomyelitis, endocarditis, toxic shock syndrome, bacteremia, and sepsis [4]. *S*. *aureus *causes both community-acquired (CA) and nosocomial/hospital-acquired (HA) infections [5]. 

*S*. *aureus *has been noted to develop multi-drug resistance. The development of antibiotic resistance started with penicillin, due to its indiscriminate use during and after the second world war. Synthetic penicillins like the methicillin and nafcillin were introduced to combat penicillin resistance. Later, during the late 1960s, the *S*. *aureus *strains resistant to the methicillin (MRSA) have emerged [2]. The MRSA strains were noted to carry specific genes (*Mec *A), which make them multi-drug resistant [6]. MRSA strains were noted to develop resistance to multiple antibiotic groups that include the aminoglycosides, cephalosporins, quinolones, and others. Therefore, vancomycin was considered as the drug of choice to treat serious infections caused by MRSA strains. 

*S*. *aureus *strains moderately resistant/intermediately sensitive to the vancomycin (VISA) had first emerged from Japan in the year 1997, followed by the first report of the occurrence of vancomycin-resistant *S*. *aureus *(VRSA) from the USA in the year 2002. There are several reports of reduced vancomycin susceptibility/vancomycin resistance emerging from throughout the world [7]. 

Linezolid, tigecycline, daptomycin, quinupristin, dalfopristin, ceftobiprole, iclaprim, and novel glycopeptides like the dalbavancin, telavancin, and oritavancin are a few choices of antibiotics to treat infections caused by the VRSA isolates [8]. Daptomycin is a cyclic lipopeptide derived from the bacterium, *Streptomyces roseosporus*, as a fermentation product [9]. The precise mechanism of action of daptomycin is not completely understood. It is known to act at the cytoplasmic membrane, binding to the membrane via a calcium-dependent insertion of its lipid tail and be an effective antibiotic against gram-positive cocci.

The laboratory identification of the MRSA and the VRSA isolates assumes increased significance for the better management of the patients. The revised Clinical Laboratory Standards Institute (CLSI) guidelines define that an *S*. *aureus *strain with a minimum inhibitory concentration (MIC) of ≤2 µg/ml as vancomycin-sensitive *S*. *aureus*. *S*. *aureus *with MIC between 4 and 8 µg/ml is considered as VISA strain and a strain with MIC of >8 µg/ml as a VRSA strain [10]. The criteria for characterization of vancomycin resistance are not universal throughout the world as noted by the cases with the British Society for Antimicrobial Chemotherapy (BSAC) and the European Committee on Antimicrobial Susceptibility Testing (EUCAST), both of which consider all strains with a vancomycin MIC ≤2 µg/ml as sensitive and those with MIC >2 µg/ml as VRSA [11-12].

Also, reports are emerging of the occurrence of vancomycin heteroresistance in the vancomycin-sensitive strains. The heteroresistance is defined as the occurrence of sub-populations/clones of *S*. *aureus *with increased vancomycin MICs within the vancomycin-sensitive strains [13]. *S*. *aureus *with such clones may be responsible for treatment failures and require treatment with high doses of antibiotics than the usual for better clinical outcomes in the patients. 

Therefore, it is important to assess the vancomycin MICs of the isolates of *S*. *aureus *for the better management of the patients. The present study is conducted to evaluate the MICs of the vancomycin and daptomycin among the MRSA isolates. 

## Materials and methods

A total of 115 MRSA isolates were collected over a period of 26 months from July 2010 to September 2012. The strains included in the study were isolated from various human clinical specimens including pus, urine, wound swabs, catheters, blood, and sputum acquired from different inpatient and outpatient departments of Prathima Institute of Medical Sciences, Karimnagar, Telangana, India. The study was conducted as a part of M.D. (Doctor of Medicine) Microbiology dissertation and was approved by the institutional ethical committee. 

*S*. *aureus *isolates were identified using standard microbiological techniques. Cefoxitin (30 µg) disk diffusion method was used to confirm the MRSA strains [10]. 

Detection of vancomycin susceptibility by agar dilution method

Vancomycin compound in pure form was obtained from Sigma Aldrich (Germany) company. A potency of 1 mg containing 1000 µg was used in the present study for the determination of MIC with the agar dilution method according to the procedures recommended by the CLSI [10]. 

Vancomycin base of 0.625 µg, 1.25 µg, 2.5 µg, 5 µg, 10 µg, 20 µg, 40 µg, 80 µg, 160 µg, 320 µg, and 640 µg was weighed using an electronic balance. The weighed amount was diluted in 0.5 ml of phosphate buffer and transferred to the flasks containing 10 ml of molten brain heart infusion (BHI) agar at 45˚C and thoroughly mixed. The media was then dispensed into different petri plates to get a final concentration of vancomycin at 0.0625 µg/ml, 0.125 µg/ml, 0.250 µg/ml, 0.5 µg/ml, 1 µg/ml, 2 µg/ml, 4 µg/ml, 8 µg/ml, 16 µg/ml, 32 µg/ml, and 64 µg/ml. These plates were later spot inoculated with the test MRSA isolates and were incubated at 35-37 °C for 16-18 hours. Results were read as the presence or absence of growth, where MIC of a strain is considered as the lowest concentration of the antibiotic at which there is no visible growth. 

Daptomycin susceptibility

An Epsilometer strip (E-strip) impregnated with daptomycin in a concentration gradient of 0.016 µg/ml to 256 µg/ml was used to determine the daptomycin MICs of VRSA isolates. 

An inoculum consisting of 0.5 McFarlands standards was prepared in peptone water. A sterile non-toxic cotton swab on a wooden applicator is dipped into the standardized inoculum, and the soaked swab was rotated firmly against the upper inside wall of the test tube to remove excess inoculum. Mueller Hinton Agar was used to make the lawn/carpet culture of the inoculum. The E-strip is placed in the center of the agar plate using an applicator. The plates were then incubated at 35-37 °C for 16-18 hours. Bacterial growth is inhibited at higher concentrations with wider zones of inhibition and as the concentration of the antibiotic decreases the zone sizes narrow down showing a tapering end. 

All the tests were run along with the standard/control strain *S. aureus* ATCC (American Type Culture Collection) 25923.

## Results

Among 115 MRSA* *isolates included in the study, 90 (78.3%) were from inpatients and 25 (22.7%) were from the outpatients. Of the 115 MRSA isolates, 13 (11.31%) belonged to the age group of 0-10 years, 19 (16.52%) were in the 11-20 years group, 16 (13.91%) in the 21-30 years age group, 19 (16.52%) in the 31-40 years group, 13 (11.31%) in the 41-50 years group, 20 (17.39%) in the 51-60 age group, and 15 (13.04%) above 60 years of age.

The isolates included in the study were acquired from the patient's attending the surgery (27%), medicine (18%), pulmonology (14.7%), pediatrics (13%), gynecology (9.5%), orthopedics (8.7%), and dermatology (8.7%) departments. *S*. *aureus *was predominantly isolated from pus samples 56 (48.70%), followed by blood cultures 28 (24.35%), sputum 18 (15.65%), and urine 10 (8.70%). Two (1.74%) isolates were from ear discharge and 1 (0.86%) was from bronchial washings.

Out of the 115 strains of MRSA included in the study, seven (6.08%) were resistant to vancomycin (VRSA) by the revised CLSI guidelines (>8 µg/ml). The MICs of 47.8% test strains ranged from 0.5 µg/ml to 2 µg/ml. There were many strains (46.08%) with increased MICs ranging from 4 µg/ml to 8 µg/ml (VISA). The detailed MIC values of all the MRSA isolates are shown in Table 1.

**Table 1:** Minimum inhibitory concentrations of MRSA isolates at different vancomycin cut-off values MRSA: Methicillin-resistant *Staphylococcus aureus*; MIC: Minimum inhibitory concentration

Vancomycin MIC (µg/ml)	Number of MRSA isolates tested (n)	Percentage (%)
0.0625	0	0
0.125	0	0
0.25	0	0
0.5	5	4.3
1	35	30.4
2	15	13
4	29	25.2
8	24	20.8
16	5	4.3
32	2	1.7
64	0	0

## Discussion

*S*. *aureus *is a normal commensal found on the skin, mucous membrane, and occasionally in the intestinal, genito-urinary, and upper respiratory tracts of healthy people. It can colonize an individual for both short (transient colonization) and extended periods (permanent colonization). It has been estimated that 30% of the world's population could be colonized with *S*. *aureus*.

It is an opportunistic pathogen and has long been known as a common cause of human infections. Although *S*. *aureus *is a part of normal human flora, it can cause a wide range of diseases, ranging from relatively mild skin infections to serious diseases. Many of these infections can rapidly become life-threatening if not treated and managed appropriately. 

In the presence of risk factors that include the extremes of age, invasive *S*. *aureus *infection like bacteremia may occur. The bacteremia may then disseminate the bacterium to distant body sites leading to endocarditis, osteomyelitis, abscess of the skin, subcutaneous tissue, lung, liver, kidney, and brain [14].

Emergence and spread of MRSA, both the CA-MRSA and the HA-MRSA, have been increasingly responsible for treatment failures as noted by a previous research report [15]. Vancomycin is the drug of choice to treat invasive MRSA infections [16]. Increased and indiscriminate use of vancomycin over the years could have been responsible for the emergence of vancomycin resistance among the *S*. *aureus *isolates. The occurrence of VRSA* *strains was noted for the first time in the USA [17]. 

Detection of the vancomycin susceptibility patterns of the *S*. *aureus *by routinely used Kirby-Bauer disk diffusion method and by the automated instruments appears very tricky as compared to identifying the MICs using the agar dilution and broth microdilution methods, which are considered more reliable and near accurate [18]. This may be attributed to the complex and manual procedures adopted, where human errors cannot be completely ruled out. 

In the present study, we have performed the agar dilution method to identify the vancomycin MICs of the 115 MRSA isolates acquired from various human clinical specimens. The study found a VRSA prevalence rate of 6.08%. The study also observed that there were several strains of *S*.*aureus *with the MIC ranges between 4 and8 µg/mL. These strains could, in the later years may develop into the VRSA strains. Also, the physicians treating infections with the VISA and the VRSA strains should either choose to modify the dosage or choose a better antibiotic. 

In a report from Nepal, the vancomycin MIC range among the MRSA isolates was noted to be between 0.0625 μg/ml and 64 μg/ml. None of the MRSA strain was VISA or VRSA* *[19]. In two other studies from Nepal which were reported two years earlier to the previously mentioned study, the vancomycin MICs were found to be in the range of 0.5-2μg/ml and 0.5-4 μg/ml with four VISA and no VRSA [20-21]. These studies reported from different geographic regions of the same country signify the complex epidemiology of VISA and VRSA strains. 

A study from China, which included 553 MRSA isolates, reported an average vancomycin MIC range of 0.38 to 3.00 μg/ml and this study had identified 11 strains of VISA (MIC>4 μg/ml) [22]. 

A Pakistan study that evaluated the vancomycin MICs among the MRSA isolates had reported that 8.8% isolates were VISA with MIC range of 4-8 μg/ml [23]. This study found no VRSA strains and observed that 68.3% isolates revealed a MIC within the range of 0.5-2 μg/ml, a similar observation as noted by the results of the present study. In a much recent study reported from Pakistan, the vancomycin resistance among *S*. *aureus *isolates was noted to be 44%, and all the resistant isolates had a MIC >256 μg/ml [24]. The same study performed *Van*A gene detection among 30 VRSA strains and found it in 46.6% VRSA isolates. An abnormally increased MIC and high prevalence rate of VRSA noted in this study should be seriously considered. 

In a study reported from Bangladesh, vancomycin MICs among the MRSA isolates ranged from 0.25 µg/ml-8 µg/ml [25]. Although the study claimed that none of the isolates were VRSA, the vancomycin MIC range reported clearly demonstrates that there could be VISA strains. 

In a report from India, it was observed that 40% of the MRSA isolates showed vancomycin MIC >3 μg/ml [26]. The same study had noted that the percentage of *S*.*aureus *with vancomycin MIC >2 μg/ml doubled from 6.1% in 2015 to 12.9% in 2016. Another study from India which assessed the vancomycin MICs in 98 MRSA isolates found that 93% isolates were in the range of 0.5-2 μg/ml, and only one strain was showing a MIC >4 μg/ml [27]. A study by Menezes et al., from Puducherry, South India, which tested 102 MRSA strains found only one strain with MIC >5 μg/ml (VISA) [28]. Another study from Hyderabad, South India tested 358 *S*. *aureus *isolates and found 16 (4.4%) isolates with an MIC range of 4-8 μg/ml (VISA), and seven (1.9%) isolates with an MIC range of 16-64 μg/ml (VRSA) [29]. Only this Indian study result appeared similar to the results of the present study which revealed a VRSA rate of 2.3%. 

A study from New Jersy, USA, which evaluated the vancomycin MIC among 166 MRSA isolates found 54% isolates had MIC <2 μg/ml and the rest 45% isolates had an MIC of 2 μg/ml, confirming the absence of both VISA and VRSA according to the CLSI guidelines [30]. 

## Conclusions

The results confirm the occurrence of increased vancomycin MICs among the MRSA isolates. The prevalence rates of VISA, VRSA, and the MICs differ significantly with the geographical regions studied. Clinical microbiology laboratories should, therefore, regularly evaluate the vancomycin MICs and the physicians must carefully adjust the treatment doses to improve the clinical outcome among the patients. Daptomycin MICs were significantly lower, highlighting its use in treating VRSA cases. 
